# *Hericium erinaceus* Improves Mood and Sleep Disorders in Patients Affected by Overweight or Obesity: Could Circulating Pro-BDNF and BDNF Be Potential Biomarkers?

**DOI:** 10.1155/2019/7861297

**Published:** 2019-04-18

**Authors:** Luisella Vigna, Federica Morelli, Gianna M. Agnelli, Filomena Napolitano, Daniela Ratto, Alessandra Occhinegro, Carmine Di Iorio, Elena Savino, Carolina Girometta, Federico Brandalise, Paola Rossi

**Affiliations:** ^1^Occupational Medicine Unit, Clinica del Lavoro Luigi Devoto, Obesity and Work Center at IRCCS Foundation Policlinico Hospital of Milan, 20133, Italy; ^2^Biochemical and Microbiology Unit, IRCCS Foundation Policlinico Hospital of Milan, 20122, Italy; ^3^Department of Biology and Biotechnology “L. Spallanzani”, University of Pavia, 27100, Italy; ^4^Department of Earth and Environmental Sciences, University of Pavia, 27100, Italy; ^5^Department of Fundamental Neurosciences (NEUFO), University of Geneva, 1211, Switzerland

## Abstract

Epidemiological data indicate that subjects affected by obesity have an increased risk of developing mood disorders. The relationship between obesity and mood disorders is bidirectional. We assessed whether a* Hericium erinaceus* treatment improved depression, anxiety, sleep, and binge eating disorders after 8 weeks of supplementation in subjects affected by overweight or obesity under a low calorie diet regimen. Looking for a possible clinical biomarker, we assessed the serum balance between brain-derived neurotrophic factor (BDNF) and its precursor pro-BDNF before and after* H. erinaceus* supplementation. Seventy-seven volunteers affected by overweight or obesity were recruited at the offices of the Department of Preventive Medicine, Luigi Devoto Clinic of Work, Obesity Centre, at the IRCCS Foundation Policlinico Hospital of Milan (Italy). Patients were recruited only if they had a mood and/or sleep disorder and/or were binge eating as evaluated through self-assessment questionnaires. We used two different enzyme-linked immunosorbent assays kits to discriminate circulating levels of pro-BDNF and BDNF. Eight weeks of oral* H. erinaceus *supplementation decreased depression, anxiety, and sleep disorders.* H. erinaceus *supplementation improved mood disorders of a depressive-anxious nature and the quality of the nocturnal rest.* H. erinaceus *increased circulating pro-BDNF levels without any significant change in BDNF circulating levels.

## 1. Introduction

Epidemiological data indicate that obesity is mutually associated with mood disorders [1–3]. Although direct evidence for brain-derived neurotrophic factor (BDNF) in the link between obesity and mood disorders has not been established, its involvement in both conditions makes BDNF an important candidate [4]. BDNF is a member of the family of neurotrophins that controls survival, growth, neurogenesis, and differentiation of a variety of neurons, particularly those in the hippocampus [5, 6]. BDNF is synthesized as the precursor protein pro-BDNF, which is cleaved by different proteases to produce the mature form. Pro-BDNF plays an important role in multiple physiological processes and shows different partial effects compared to mature BDNF, which activates apoptotic pathways in neurons and glia [7]. Pro-BDNF exerts its effect by binding to the p75 neurotrophin receptor [8], whereas BDNF acts via TrkB receptors, which are widely expressed in various part of the brain [9, 10]. Defects in the mechanism of conversion of pro-BDNF into BDNF or an altered balance of the two forms have been linked with cognitive impairment, psychiatric disorders [11–13], and anxiety-like behaviours [14].

The neurogenic hypothesis of depression was formulated based on the demonstration that neurogenesis is negatively regulated by stressful experiences and positively regulated by treatment with an antidepressant. Antidepressant drugs or other treatments for mental disorders can restore the deficient peripheral and brain levels of BDNF and stimulate adult neurogenesis [4, 15]. Among neurogenic zones in the adult brain, the hippocampus is involved in higher cognitive function, memory processes, and affective behaviour [16].

Chronic consumption of a high-fat diet leads to obesity. The consequent chronic systemic inflammation, possibly through a change in the gut, could influence the mood and behaviour of the host [2].

Chronic consumption of a high-fat diet in mice is associated with reduced cerebral BDNF levels in the reward circuitry [2] and reduced neurogenesis in the dentate gyrus [17–20]. Dietary restrictions have the opposite effect of increasing the expression of hippocampal BDNF and promoting survival of newly generated neurons [21]. Despite data on the cerebral BDNF level in a specific region of the brain in preclinical models, no direct evidence indicates a causal relationship between BDNF, the induction of adult neurogenesis, and mood disorders [4].

A validated animal model to study the pathogenesis and the efficacy of treatment of major depressive disorder is felt useful [22, 23]. The forced swim test (FST) is one of the most used common tests in rodents to address the extent of a depression like behaviour. The current state of the art is that certainly FST is a powerful paradigm to study the mechanism underlying coping with inescapable stressors [24]. The management of coping with them is obtained by processing in prefrontal cortical circuitry and glucocorticoid feedback [24].

Meta-analyses of clinical studies based on measurements of peripheral BDNF have reported significantly lower BDNF levels in patients with major depression, schizophrenia, bipolar disorder, or autism spectrum disorder (for meta-analyses see [25–28]).

However, severe discrepancies among the studies were highlighted, which even reported opposite results (increase or no change in peripheral BDNF levels) [29–31]. In particular, Molendijk and colleagues suggested that low serum BDNF levels are evident during depression and normalize during remission [32]. The effect of some antidepressant in the increased serum level of BDNF does not parallel clinical improvements such as the severity of depression symptoms [32].

The Netherlands Study of Depression and Anxiety (NESDA), a longitudinal cohort study on the long-term course and consequences of depression and anxiety, complicates even more the picture. In the study the results after a follow-up of 2 years in nondepressed controls, incident-depressed, persistent depressed, and remitted patients are reported [33]. Persistent depressed and remitted patients showed a steeper decrease of BDNF serum levels over time compared with nondepressed controls [33]. The baseline BDNF serum level at the beginning of the study among the different cohorts was not different [33].

Many studies have demonstrated a relationship between human obesity and a decrease of serum BDNF level (for a systematic review of the literature and a meta-analysis see [34]). Among its many functions, BDNF exerts an anorexigenic effect, mediated by the mammalian target of rapamycin (mTOR) [9]. Activated mTOR increases lipid and protein synthesis and inhibits protein degradation in the hypothalamus. Deleting BDNF or a BDNF knockdown in the brain or hypothalamus induces hyperphagia and obesity in mice [9]. Furthermore, plasma levels of BDNF are inversely correlated with fasting plasma glucose levels, suggesting a potential role for BDNF in glucose homeostasis and diabetes [35].

The hypothesis that BDNF is a candidate link between obesity and mood disorders is further supported by findings that the cerebral level of BDNF expression is under the influence of the gut microbiota, and a dysbiotic experimentally induced microbiota in mice leads to reduced hippocampal BDNF gene expression [36].

Given this complex framework, the use of circulating BDNF as biomarker in clinical studies is questioned by the presence of discordant results [37, 38], which are likely due to the variety and lack of antibody specificity as they do not selectively recognize pro-BDNF and BDNF [31, 39].

*Hericium erinaceus, *also known as Yamabushitake or Lion's Mane, is an exceptional health-promoting species.* H. erinaceus *has a neuroprotective role in neurodegenerative diseases,* in vitro* and in preclinical studies [40–42]. The* H. erinaceus* fruiting body and mycelia contain an exceptionally large amount of structurally different bioactive components, including polysaccharides, erinacines, hericerins, steroids, alkaloids, and lactones that play roles preventing, alleviating, or treating major diseases, including cancer, depression, diabetes, lipidaemia, and neurodegenerative diseases [43, 44]. Hericenones and erinacines of* H. erinaceus* cross the blood-brain barrier and increase the synthesis of trophic factors, such as nerve growth factor and BDNF [45–48].

Our previous data are in agreement with the hypothesis of increased neurogenesis in the hippocampal dentate gyrus after* H. erinaceus* supplementation in wild-type mice [49]. Moreover, many studies have established that new-borne neurons in the dentate gyrus are required for mediating some of the beneficial effects of antidepressant treatment [50–52].

Furthermore, a study in mice reported reduced expression of BDNF and TrkB in the hippocampal region after restraint stress and treatment with* H. erinaceus* restored the brain BDNF levels [47, 48]. According to our hypothesis of increased neurogenesis in the hippocampal dentate gyrus after* H. erinaceus* supplementation, Chiu et al. [47] described the effect of erinacine A-enriched* H. erinaceus* mycelia as an antidepressant that modulates BDNF/PI3K/Akt/GSK-3 signalling in mice.

The aim of this study was to investigate the effect of* H. erinaceus* supplementation on mood disorders and its relationship with pro-BDNF and BDNF circulating levels in subjects affected by overweight or obesity to test whether the two isoforms could be potential biomarkers in clinical studies.

## 2. Materials and Methods

### 2.1. Study Design

**Figure 1:**
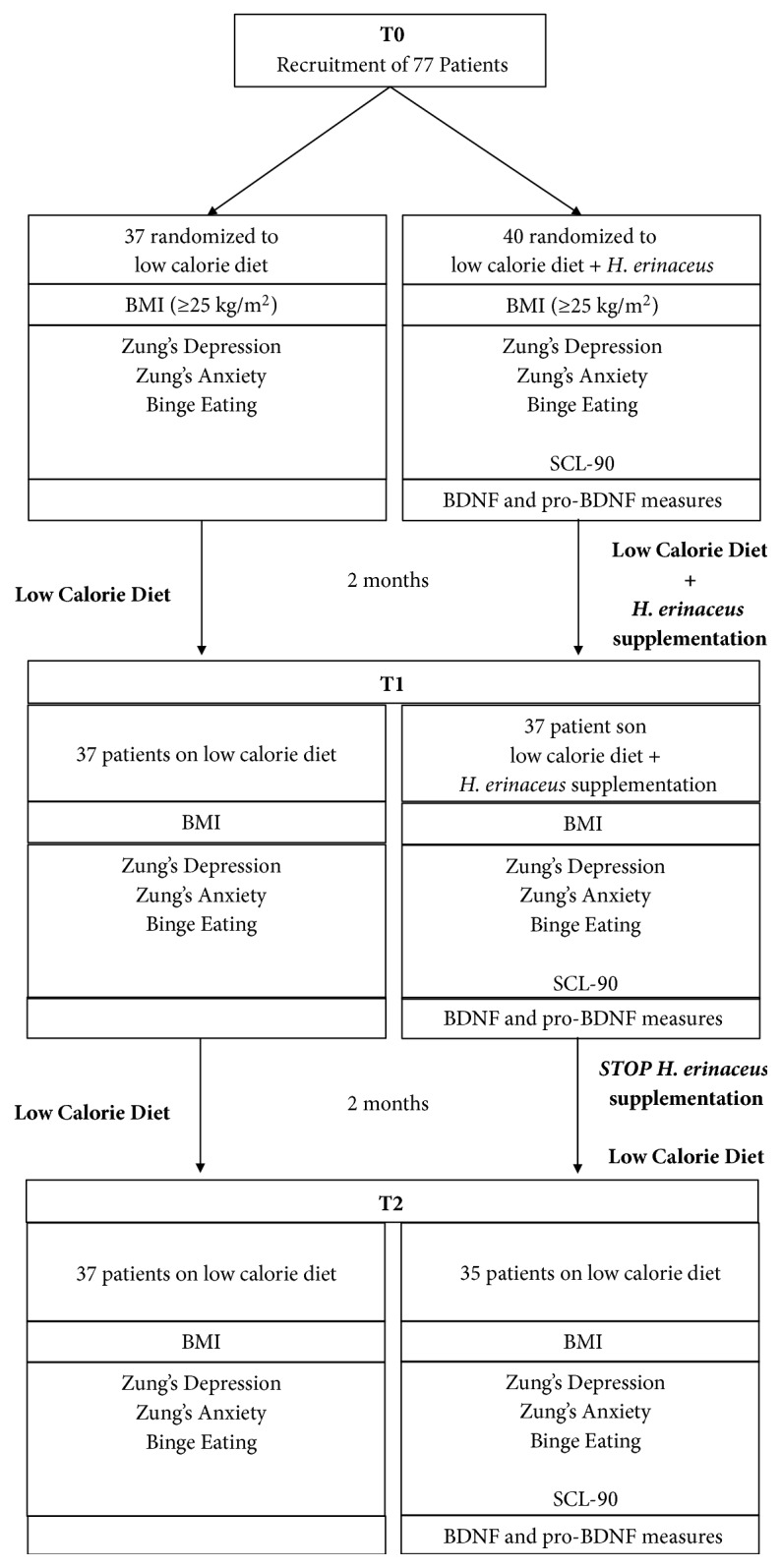
Flow diagram of progress of participants through the study.

Figure 1 summarizes the recruitment and progress of these participants through the study. The experimental plan develops with the recruitment time (T0), after* H. erinaceus *supplementation for 2 months (T1) and the last sampling time (T2) after the 2 months wash-out.

Seventy-seven volunteers (62 females and 15 males) with a body mass index (BMI) ≥ 25 Kg/m^2^ (age 53.2 ± 0.7 years old) were recruited from the Department of Preventive Medicine, Luigi Devoto Clinic of Work, Obesity Centre, at the IRCCS Foundation Policlinico Hospital (Milan, Italy). Upon entering the study, each participant signed an informed consent form and provided detailed information about their general health, dietary intake, and lifestyle. This research was carried out in accordance with the principles stated in the Declaration of Helsinki for Research on Human Subjects and was approved by the local ethics committee (study registration number: 1370). The following exclusion criteria were considered: presence of therapy with antidepressant and/or anxiolytic drugs, mushroom allergies, pregnancy, language barrier, and previous organ transplantation. We included only patients with one or more mood disorders, evaluated by Zung's Depression Self-Assessment Scale [53, 54], Zung's Anxiety Self-Assessment Scale [55], Symptom Checklist-90 (SCL-90) [56], and the binge eating scale (BES) [57]. The patients were invited to participate in the study through an individual interview during which the study objectives were presented. All participants were randomly included in a control group (n = 37) or an* H. erinaceus* intervention group (n = 40). All volunteers who participated in the study followed a low calorie diet. All participants were given a diet of 1400 kcal for women and 1700 kcal for men (CHO 52%, Lipids 30%, and protein 18%). The subjects underwent a monthly clinical follow-up where diet adherence and anthropometric parameters were checked by a skilled dietitian. BMI changes in the two experimental groups at T0, T1, and T2 are reported in Table 1. It should be noted that there is no difference in the two experimental groups and therefore the BMI decrease reflects the low calorie diet regimen.

Patients in the intervention group received three capsules/day for 8 weeks of an* H. erinaceus* dietary supplement (*“Micotherapy Hericium*”) provided by A.V.D. Reform s.r.l. (Noceto, Parma, Italy).

The supplement composition was 80% bulk mycelia and 20% fruiting body extract (Table 2). Extraction conditions from fruiting bodies were the following: biomass/solvent ratio was 1/15; extraction time was 3 h. Solvents for distinct extractions were water and pure ethanol, respectively. The remaining liquid phase was dried under vacuum at 70°C and -0.9 bar and further milled by UPZ mill (Hosokawa Alpine Aktiengesellschaft, Augsburg, Germany). Final particles were mostly smaller than 100 *μ*m. Mycelial biomass was dried and milled by the same procedure.

The polysaccharide content was determined by *β*-Glucan Assay Kit (Megazyme, Ireland) and expressed as total (*α* plus *β*) glucan content. The nutritional composition of* Micotherapy Hericium *is reported in Table 3.

At the end of the treatment, the subject's compliance was checked by a daily diary that reported the number of consumed capsules. A safety assessment questionnaire was completed by all subjects. The subjects were evaluated at three experimental times of T0, T1, and T2 as described in Figure 1.

In the* H. erinaceus* group, we recorded three drop-outs at T1 and five drop-outs at T2, while in control group we had no drop-outs.

### 2.2. Self-Evaluation Scales

In the normal clinical routine, the effect of hypocaloric diet on mood disorders, such as depression, anxiety, and binge eating disorders, was investigated by using the Zung Self-Rating Depression [58] and Anxiety [59]. Scale and Binge eating scale (BES). In addition, in* H. erinaceus *group the effects on mood disorders were also investigated by using an Italian version of the SCL-90 [60]. SCL-90 is considered a psychometrically more refined tool than Zung's tests.

The SCL-90 differs from other psychiatric questionnaire as it measures both externalizing (such as impulsivity, hostility, and aggressiveness) and internalizing (such as depression, anxiety, sleep, and somatizations) symptoms. In clinical practice, the SCL-90 is considered a more articulated psychometric test and is frequently adopted as an outcome measure in psychotheraphy [61] and primary care settings [62]. By using SCL-90 and Zung's tests, we obtained further data about symptomatic dimensions indicative of anxiety, depression, and sleep disorders.

Subjects were recruited only if they tested positive to one or more administered test.  Table 4 reports the range values to define the entity of the disorder, such as “low”, “moderate”, and “high”.

### 2.3. Zung's Depression Scale

Zung's Self-Rating Depression Scale is considered the prototype of the self-assessment scales and allows us to obtain a fast and quantitative evaluation of psychological (10 items), affective (2 items), and somatic (8 items) symptoms of depression in patients. The patient must evaluate the frequency of appearance of the symptom described in the item on a scale from 1 to 4 (“rarely”, “sometimes”, “often”, and “almost always”) and the obtained scores were summed. We considered a patient positive for depression with a score equal or greater than 44 for Zung's Depression Self-Assessment Scale (Table 4).

### 2.4. Zung's Anxiety Scale

This scale consists of 20 items, and the evaluation is exactly as described for Zung's depression scale, ranging from 1 to 4. For 5 items (numbers 5, 9, 13, 17, and 19) on exploring welfare, the score value was opposite to the other 15 items addressing anxiety symptoms. In this way, the risk that the patient mechanically provides the same score to all items was reduced. We considered a patient positive for anxiety with a score equal or greater than 41 for Zung's Anxiety Self-Assessment Scale (Table 4).

### 2.5. BES

The BES includes 16 groups of statements, and the patient chooses the statement that is closest to describing their own emotional feelings. A value from 0 to 3 is assigned to each statement. The total score is obtained by summing all of the values. A range between 10 and 16 indicates the presence of binge eating. A score > 17 indicates a high risk for a food disorder. We considered a patient positive for binge eating with a score equal or greater than 10 for the BES (Table 4).

### 2.6. SCL-90

Due to its easy use and the wide range of symptoms explored, the SCL-90 is employed as a clinical and research screening and monitoring tool, thus arriving to cover almost entirely the psychopathological spectrum. Therefore, it is used as an outcome measure in research in psychotherapy and in basic medicine settings. The SCL-90 is composed of 90 items, and patients provide an evaluation from 0 (“not at all”) to 4 (“very much”). The results identify ten different symptomatic dimensions, among which those related to “depression” (13 items), “anxiety” (10 items), and “sleep disorders” (3 items) appear. For each dimension, we calculated the mean score value. We considered a patient positive for a mood disorder with a score equal or greater than 1 for the SCL-90 (Table 4).

### 2.7. Determination of Pro-BDNF and BDNF Serum Levels by Direct Enzyme-Linked Immunosorbent Assay (ELISA) 

Blood samples were collected after an overnight fast, and the serum was immediately isolated and stored at −20°C. To avoid seasonal variations and correlation with the amount of ambient sunlight we collected serum between June and December [63].

By using specific antibodies, serum pro-BDNF and BDNF levels were measured in patients of the* H. erinaceus* intervention group (n = 10) by using two different competitive ELISA immunoenzymatic colorimetric kits (for pro-BDNF, SK00752-09, and BDNF SK00752-01 provided by Aviscera-Bioscience, Santa Clara, CA, USA). The detection range for BDNF was 23–1500 pg/ml (sensitivity 5–8 pg/ml and intra-assay precision 4–6%). The detection range for pro-BDNF was 0.78–25 ng/ml (sensitivity 0.25 ng/ml and intra-assay precision 4–6%). The protocols were performed according to the manufacturer's instructions. Optical density was measured using an automated microplate reader (ELx808; BioTek, Winooski, VT, USA).

### 2.8. Statistical Analysis

After using Bartlett's test for homogeneity of variance, one-way repeated-measures analysis of variance (ANOVA), two-way ANOVA, and Student's* t*-test were used to compare the groups. Descriptive statistics are reported as mean ± SEM. A p value < 0.05 was considered significant. Statistical analyses were performed with GraphPad Prism 7.0 software (GraphPad Software Inc., La Jolla, CA, USA).

## 3. Results and Discussion

### 3.1. Results

#### 3.1.1. Mood Disorders by Zung's Tests and Binge Eating Scale

At the recruitment (T0), all patients were tested for quantitative evaluation of the psychological symptoms according to Zung's depression scale, Zung's anxiety scale, and the BES (Table 5). Notably, a high binge eating score was present in 21.6% of patients affected by overweight or obesity, whereas high depression and anxiety scores were present in only 0-5% of patients. Moderate depression, anxiety, and BES were present in 32–39.3% of patients. The remaining patients were distributed in the low anxiety, low or very low depression, and low BES score (Table 5).

**Figure 2:**
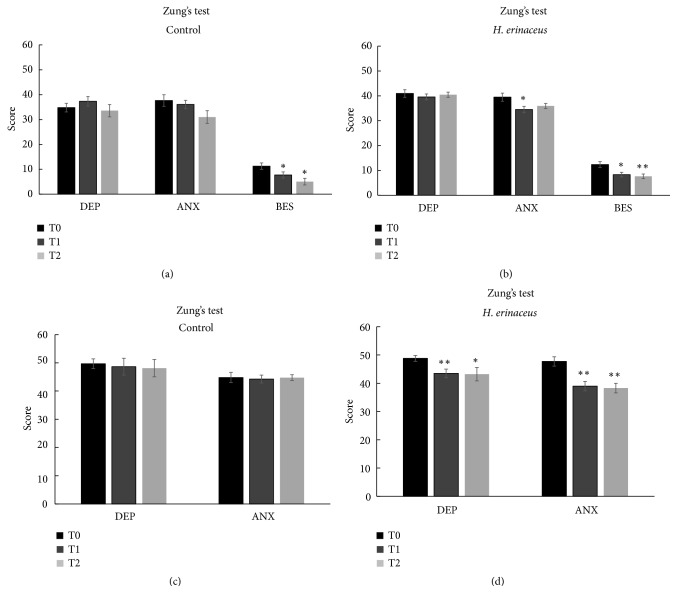
Mean values ± SEM of Zung's depression (DEP), Zung's anxiety (ANX), and binge eating scale (BES) tested by self-evaluation: (a) control and (b)* H. erinaceus* groups before (T0), after (T1), and in* H. erinaceus*. (c) Patients in control condition selected for symptomatology in T0, T1, and T2. (d) Patients selected for symptomatology before (T0), after (T1), and in* H. erinaceus* wash-out condition (T2). *∗*p<0.05 and *∗∗*p<0.01 were obtained by the comparison versus T0 in any experimental group (according to two-way ANOVA). The comparison of values between T1 and T2 does not show any statistically significant differences.

Figure 2 shows the mean score values for Zung's depression scale, Zung's anxiety scale, and the BES disorder at different experimental times in the control (Figure 2(a)) and* H. erinaceus* (Figure 2(b)) groups. No significant differences were observed between the score mean values obtained in the control and* H. erinaceus* groups for depression. Interestingly, after* H. erinaceus* supplementation, we measured a significant improvement in anxiety disorder of about 12.6%.

Notably, the BES score decreased significantly in the control (Figure 2(a)) and after* H. erinaceus* supplementation (Figure 2(b)) (percentage of decrease: 31.5% versus 32.7% at T1 and 55.5% versus 38.2% at T2 in control and* H. erinaceus* groups, respectively).* H. erinaceus *did not exert any additional effect on the BES, suggesting that the improvement was due to the low calorie diet regimen.

We selected patients in control group positive for mood disorders at the recruitment time (n=14 for depression and n=15 for anxiety) and we verify the effect of the low calorie diet alone (Figure 2(c)). Any statistically significant difference in mean value score for mood disorders was recorded.

Furthermore, we verified the effect of* Hericium erinaceus* supplementation only in patients positive for depression (n=16) and anxiety symptomatology (n=15, Figure 2(d)) at recruitment time (T0).

Regarding depression mood disorders at T0, among the recruited patients randomized to receive low calorie diet and* H. erinaceus, *40% of them fell in moderate, and 7.5% in high degree in Zung's depression scale (mean score value 48.8 ± 1.03). After 2 months of* H. erinaceus *(T1) supplementation the mean score value decreased to 43.5 ± 1.54, indicating a low degree depression symptomatology. The value remained stable after* H. erinaceus* wash-out (T2, mean score value 43.2 ± 2.38).

Regarding anxiety mood disorders at T0, among selected patients for anxiety symptomatology 42.5% of them fell in moderate and 2.5% in high degree in Zung's anxiety scale (mean score value 47.7 ± 1.66). After 2 months of* H. erinaceus *supplementation (T1) the mean score value decreased to 39 ± 1.68, indicating a low degree anxiety symptomatology. The value remained stable after* H. erinaceus* wash-out (T2, mean score value 38.3 ± 1.68).

#### 3.1.2. Mood Disorders by SCL-90 Test

**Figure 3:**
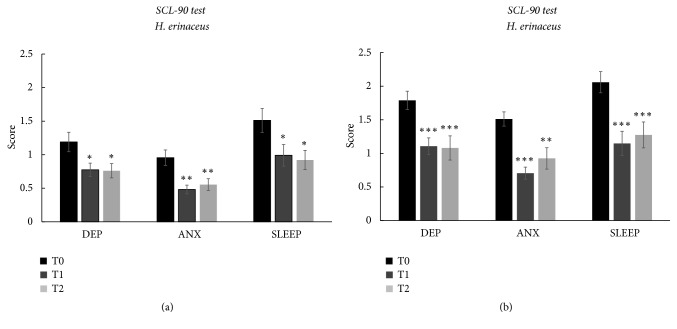
(a) Mean score values ± SEM obtained by means of SCL-90 in* H. erinaceus* group before (T0), after (T1), and in* H. erinaceus* wash-out condition (T2) in (DEP), anxiety (ANX), and sleep disorders (SLEEP). (b) Patients selected for symptomatology before (T0), after (T1), and in* H. erinaceus* wash-out condition (T2). The *∗*p<0.05 and *∗∗*p<0.01 obtained by the comparison versus T0 (according one-way ANOVA, Tukey post hoc test). The comparison of values between T1 and T2 does not show any statistically significant differences.

In the intervention group, we further investigated the effect of the* H. erinaceus *supplement on depression, anxiety, and sleep disorders using the SCL90 (Figure 3).

Depression, anxiety, and sleep disorders decreased significantly according to the SCL-90 after 8 weeks of oral supplementation with* H. erinaceus *(Figure 3(a)). In particular, depression symptomatology decreased by 34.9% and 36% at T1 and T2, respectively. As concerned anxiety symptomatology, after* H. erinaceus* supplementation, we measured a score decrease by 49.6% at T1 and it was maintained at 41.9% at T2. Sleep disorders improved 34.4% at T1 and decreased by 39.1% at T2. Notably, there was no wash-out effect after 8 weeks of the* H. erinaceus *supplementation because the improvements in depression, anxiety, and sleep disorders were maintained.

It should be noted that patients showed a positive score for depression and sleep disorders at recruitment time (T0), after* H. erinaceus* supplementation fell down to a low score value.

Furthermore, as shown before for Zung's tests, we verified the effect of* H. erinaceus* supplementation only in patients positive for depression (n=19), anxiety (n=18), and sleep disorders (n=27, Figure 3(b)) at recruitment time (T0).

By selecting patients for symptomatology, we obtained an even clear picture of the* H. erinaceus* effect on mood disorders (Figure 3(b)). After* H. erinaceus* supplementation, depressed patients on the mean fell close to 1 score value, that is, the limit value to recognize depression symptomatology. The effect was still evident after 2 months of* H. erinaceus* wash-out (T2). For anxiety disorder, the* H. erinaceus* effect is more evident and patients fell under the limit value for anxiety disorder. Furthermore, patients with sleep disorders moved from a very high value score of 2.06 ± 0.16 to 1.15 ± 0.18 after* H. erinaceus* supplementation and the effect remained at T2 with a mean value score of 1.27 ± 0.19.

Sleep disorder shows a mean reduction of 44.25% at T1 and 38.13% in wash-out condition (T2).

#### 3.1.3. Mood Disorders by Combined Analysis

**Figure 4:**
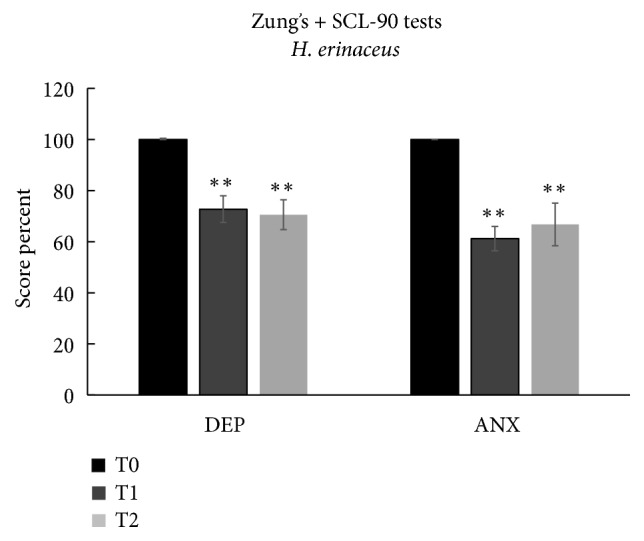
Patients selected for symptomatology positive at T0. Depression (DEP), anxiety (ANX), and sleep scale's scores (mean ± SEM) at T0, T1, and T2. *∗∗*p<0.01 indicates the comparison at T1 and T2 versus T0 (according to one-way ANOVA, Tukey post hoc test). The comparison of values between T1 and T2 does not show any statistically significant differences.

We further analyzed the effects of* H. erinaceus* by collecting the patients according to mood disorder symptomatology at T0 (Figure 4) evaluated by the combined analysis of Zung's and SCL-90 tests for depression (n = 22) and anxiety (averaged data, n = 20). Data obtained by Zung's and SCL-90 tests were normalized to the value obtained before* H. erinaceus* supplementation (T0).* H. erinaceus* reduced depression by 27.2% at T1 and by 29.4% at T2 and reduced anxiety by 38.8% at T1 and by 33.2% at T2. All reductions were significantly different from the scores at T0.

These data highlight the effectiveness of* H. erinaceus* for significantly reducing depression and anxiety. It should be noted that all subjects moved from a score index of the “moderate state” to the “low state” for anxiety and depression after the* H. erinaceus* supplementation, and the effect remained after 8 weeks of wash-out.

#### 3.1.4. Pro-BDNF and BDNF Serum Level

**Figure 5:**
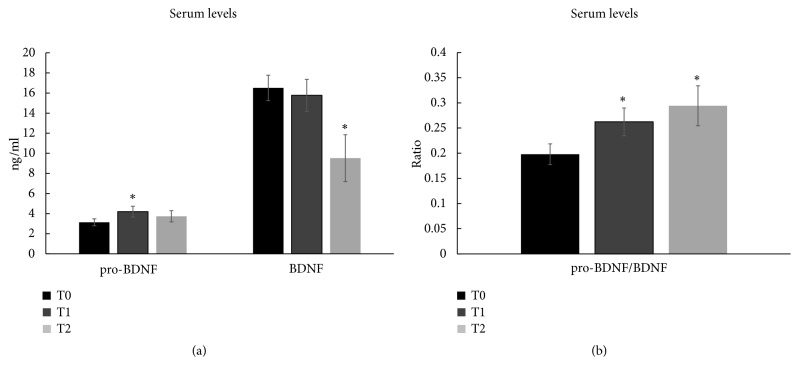
(a) Pro-BDNF, BDNF, and (b) pro-BDNF/BDNF ratio serum levels ± SEM in patients treated with* H. erinaceus* at T0, T1*, and* T2. *∗*p<0.05 indicates significant differences between different time versus T0 tested by paired Student's t-test. The comparison of values between T1 and T2 does not show any statistically significant differences.

Finally, we tested the hypothesis that* H. erinaceus* increases circulating BDNF levels, thus, allowing its use as a potential clinical biomarker. We selected patients with anxiety, depression, and sleep disorders at T0 (n = 10) and measured serum pro-BDNF and BDNF levels at T0, T1, and T2 (Figure 5). After the* H. erinaceus* supplementation, a significant increase in serum pro-BDNF level was observed at T1, without any change in serum BDNF at T1 and a significant decrease in BDNF level at T2 (Figure 5(a)). The increase in the pro-BDNF/BDNF ratio after* H. erinaceus* supplementation (Figure 5(b)) reflected the increase in pro-BDNF at T1, whereas the increase after wash-out (Figure 5(b)) reflected the decrease in BDNF at T2.

### 3.2. Discussion

Accumulating data suggest a correlation between obesity and mood disorders, such as depression, anxiety, binge eating, and sleep disorders. Seventy-seven patients affected by overweight or obesity were recruited at the Obesity Centre at IRCCS Foundation Policlinico Hospital of Milan and evaluated for mood disorders. 32% of patients showed moderate depression, 39.2% moderate anxiety, and 33.8% moderate emotional eating, whereas only 5% were highly depressed and 21.6% have high emotional eating behaviour, who were, therefore, at a high risk for a food disorder.

The present study investigated the effect of an ethanol extract of the mycelia and fruiting bodies of* H. erinaceus* on depression, anxiety, binge eating, and sleep disorders in patients affected by overweight or obesity subjected to a low calorie dietary regimen.

In a first attempt, all recruited patients were analyzed by Zung's depression and anxiety test. The mood disorders did not improve in response to the low calorie diet alone, whereas the low calorie regimen was effective for reducing symptoms related to emotional eating. After 8 weeks of* H. erinaceus *supplementation and the low calorie diet, patients showed a significant reduction in depression, anxiety, binge eating, and sleep disorders. Therefore, the improvement in binge eating was not due to* H. erinaceus* supplementation, but to the low calorie diet.

If patients are selected by symptomatology, the effect of H. erinaceus supplementation on mood disorders appears more evident. The effect of* H. erinaceus* lasted in the absence of treatment.

As suggested by scientific literature, the SCL-90 assessment questionnaire showed an increased sensitivity to discriminate anxiety and depression before and after* H. erinaceus* supplementation compared to Zung's tests.

SCL-90 test confirms the effect of* H. erinaceus* as obtained by Zung's test. In patients selected for symptomatology, after* H. erinaceus* supplementation, depression, anxiety, and sleep disorder decreased to a value above (depression) or lower (anxiety and sleep disorder) than 1.

Finally, by combining Zung's and SCL-90 tests we obtained an integrate value that returns a mean reduction of the depression score of about 30% and above 40% of the anxiety symptoms. Again, the effect of* H. erinaceus* lasted in the absence of treatment.

These data agree with previous clinical data, which described an improvement in mood disorders after* H. erinaceus *supplementation. In particular, a double-blind placebo controlled clinical trial investigated the effects of* H. erinaceus* on mild cognitive impairment [64]. The cognitive function scale improved significantly in the treatment group compared with the placebo group. Nagano et al. [65] conducted a randomized, double-blind, placebo-control trial that investigated the beneficial effects of* H. erinaceus* on depression and anxiety. An oral intake* H. erinaceus* reduced depression and anxiety in women. We previously reported that oral supplementation with* H. erinaceus *induces significant improvements in novelty-seeking behaviour and recognition memory in wild-type mice compared with controls [49, 66, 67]. Novelty-seeking behaviour is essential in all mammalian species [68] and is considered one of the six major personality behaviours. In contrast, neophobia describes a hesitance to engage with novel objects or novel places, the presence of rigid evaluative patterns, and reduced flexibility. Increased neophobia reduces novelty-seeking behaviour and is considered a risk factor for anxiety disorders and is a core symptom of depression [69, 70]. Therefore, also our data obtained in preclinical model suggested that the* H. erinaceus* treatment could decrease neophobia and consequently anxiety disorders.

The search for biomarkers in blood, serum, or plasma to support the diagnosis or monitor the efficacy of therapies is a major unmet clinical need. BDNF could be a candidate linking overweight/obesity with mood disorders. Given that BDNF passes through the blood-brain barrier, circulating peripheral BDNF should reflect central BDNF levels. Despite the data on cerebral BDNF levels in specific regions of the brain in preclinical model, the use of circulating BDNF as biomarker in clinical studies has been limited by poor reproducibility of results. This limitation is likely due to the variety of antibodies used for BDNF analyses [39], as most are unable to distinguish between the two isoforms pro-BDNF and BDNF [31, 39, 71, 72].

As previously suggested [47, 49, 67] we tested the hypothesis of an increase in BDNF after* H. erinaceus* supplementation by measuring serum BDNF levels. We used specific antibodies that recognized specific epitopes of the two different isoforms to assess the balance between pro-BDNF and BDNF before and after* H. erinaceus* supplementation.

A significant increase in serum pro-BDNF level was observed after* H. erinaceus* supplementation, without any significant change in serum BDNF. Further investigations are necessary to study the regulation of proteolytic cleavage of the pro-BDNF precursor into the mature BDNF isoform [73].

The use of specific antibodies to recognize pro-BDNF and BDNF isoforms was not resolutive to answer the severe discrepancies among studies addressing the relation between peripheral BDNF levels and mood disorders [25–33]. We cannot exclude that the lack of an association between* H. erinaceus* supplementation and circulating BDNF level might be that the majority of our patients had less severe mood disorders with moderate depression and anxiety.

Furthermore, our previous data suggest, after* H. erinaceus* supplementation, an increase in BDNF in a specific brain area, such as the hippocampus and parahippocampal area [47, 49, 67], and we do not know how and whether this increase is reflected in peripheral BDNF levels. Future studies will be addressed to this topic.

## 4. Conclusions

In conclusion,* H. erinaceus *promoted an improvement in mood disorders of a depressive-anxious nature and of the quality of nocturnal rest. These effects persisted after eight weeks of* H. erinaceus *wash-out, suggesting that* H. erinaceus *might affect neuronal plasticity as expected by a NGF or BDNF like effect. In addition, although binge eating improved, the data do not seem related to a specific effect of* H. erinaceus*. The improvement in mood disorders was associated with a change in peripheral pro-BDNF and in the pro-BDNF/BDNF ratio, suggesting that further investigation must address the role of the two neurotrophin isoforms investigated here.

This study was limited by some criticisms such as the limited number of patients and the lack of a placebo group, and, therefore, we considered it as a pilot study that necessarily should be confirmed by randomized placebo controlled trials.

## Figures and Tables

**Table 1:** BMI (mean ± SEM) of the control and *H. erinaceus *experimental groups at T0, T1, and T2. The experimental groups do not show significant differences in BMI value (tested by two-way ANOVA).

BMI [kg/m^2^]	Control	*H. erinaceus*
T0	33.36 ± 0.83	33.12 ± 0.84
T1	32.08 ± 0.88	32.01 ± 0.82
T2	31.76 ± 1.41	31.63 ± 0.72

**Table 2:** Nutrient composition of the supplement “*Micotherapy Hericium*” from A.V.D. Reform s.r.l., Noceto (Parma).

Component	mg /capsule	mg /die	g / 8 weeks
*H. erinaceus* mycelium	400	1200	67.2
*H. erinaceus* fruiting body extract	100	300	16.8
Total polysaccharides amount	50	150	8.4

**Table 3:** Nutritional composition of the supplement “Micotherapy Hericium” from A.V.D. Reform s.r.l., Noceto (Parma).

Analyte	Fruiting body extract	Mycelium powder
Kcal/g	2.23	1.98
Crude proteins %	8.25	10.22
Crude fat %	0.17	1.02
Crude fiber %	5.92	39.2
Polysaccharides/total glucans %	>45	>37
Sodium %	0.0146	0.0031

**Table 4:** Scores value and interpretation results for the different psychometric test valuated.

Zung's Depression Scale (1974)	

20-31	Very low
32-43	Low
44-55	Moderate
56-67	High
68-80	Very High

Zung's Anxiety Scale (1971)	

20-40	Low
41-60	Moderate
61-80	High

Sympton Checklist – 90 (SCL - 90) scale	

<1	Low
≥1	Score of interests about items considered (depression, anxiety, sleep disorders)

Binge eating scale (BES; 1982)	

<9	Low risk of food disorder
10-16	Emotional eating
>17	High risk of food disorder

**Table 5:** Quantitative evaluation at T0 (recruitment time) of psychological symptoms. Patients' percentage in different scale score of Zung's depression, Zung's anxiety, and binge eating.

	Very low %	Low %	Moderate %	High %
Zung's Depression	12.5	50.5	32	5
Zung's Anxiety	-	60.7	39.3	0
Binge Eating	-	44.6	33.8	21.6

## Data Availability

The data used to support the findings of this study have not been made available to protect the subjects' privacy.
